# The Discovery of Modern Anæsthesia

**Published:** 1894-03

**Authors:** 


WE have received from L. W. Nevins, New York, author and publisher, "The Discovery of Modern Anæsthesia," together with a picture, "Souvenir of the Discovery of Anæsthesia." The volume gives a brief history of the discovery of anæsthesia between the years 1842 and 1846 and speaking of the four men (two physicians and two dentists), each of whom claimed for himself the honor of being the original discoverer; the author says :

"These four men fought over the subject, and labored for the honor, until one lost his mind and committed suicide in a prison cell in the city of New York. Another died in an insane asylum. Another left his sick bed under peculiar circumstances, dying a short time after in Central Park, New York. The fourth was stricken with paralysis, while visiting a patient, and died the following day.

In the Public Garden at Boston, Mass., there stands a beautiful monument known as the 'Ether Monument,' erected to commemorate the discovery of anæsthesia. As it has never been decided who the real discoverer was, it bears no man's name but that of Thomas Lee, who gave ten thousand dollars towards its erection; but it bears upon its four sides four appropriate inscriptions." R. G.

